# Paired tumor biopsy analysis and safety data from a pilot study evaluating Tremelimumab - a monoclonal antibody against CTLA-4 - in combination with ablative therapy in patients with hepatocellular carcinoma (HCC)

**DOI:** 10.1186/2051-1426-2-S3-P98

**Published:** 2014-11-06

**Authors:** Austin Duffy, Sid P Kerkar, David E Kleiner, Susanna Ulahannan, Metin Kurtoglu, Oxana Rusher, Suzanne Fioravanti, Melissa Walker, William D Figg, Kathryn Compton, Aradhana Venkatesan, Nadine Abi-Jaoudeh, Brad Wood, Tim F Greten

**Affiliations:** 1 National Cancer Institute, National Institutes of Health, Bethesda, MD, USA; 2 Laboratory of Pathology, Center for Cancer Research, National Cancer Institute, National Institutes of Health, Bethesda, MD, USA; 3 Gastrointestinal Malignancies Section, Center for Cancer Research, National Cancer Institute, National Institutes of Health, Bethesda, MD, USA; 4 Clinical Pharmacology Program, Center for Cancer Research, National Cancer Institute, National Institutes of Health, Bethesda, MD, USA; 5 Radiology and Imaging Sciences, Center for Cancer Research, National Institutes of Health, Bethesda, MD, USA

## Background

Tremelimumab is a fully human monoclonal antibody that binds to CTLA-4 expressed on the surface of activated T lymphocytes and results in inhibition of B7-CTLA-4-mediated down regulation of T cell activation. Both transcatheter arterial chemoembolization (TACE) and radiofrequency ablation (RFA) have been shown to induce a peripheral immune response which may enhance the effect of anti-CTLA4 treatment in patients with advanced HCC.

## Methods

Patients with HCC (Childs Pugh A/B7; Barcelona Clinic Liver Cancer Stage C; ECOG 0/1; previously progressed on Sorafenib) are being enrolled in a pilot study of Tremelimumab at 2 dose levels (DL1 and DL2) until disease progression (irRECIST). Subtotal TACE or RFA is performed during study week 6 with DLT evaluation period encompassing first 8 weeks of study. Tumor tissue is collected for analysis at baseline on all patients with optional on-treatment tumor biopsies performed at the time of the radiologic procedure.

## Results

11 pts have been treated so far, 6 pts at DL1 and 5 pts at DL2; M:F 9:2; Median age = 54(range 42-75); Cirrhosis present in 7pts. Hepatitis B/C/neg: 3/6/2. 4 pts received TACE, 7 underwent RFA during week 6 of Tremelimumab therapy. Tumor tissue is being collected for analysis at baseline in all patients. Once DL1 was established as safe and feasible on-treatment tumor biopsies are being performed at the time of the radiologic procedure (Day 36 +/- 96hrs) on all patients. So far 2 of 5 patients treated at DL2 have shown extensive immune cell infiltration on tumor biopsies after 6 weeks of Tremelimumab. More in depth analysis are currently being conducted and will be presented together with safety data.

## Conclusions

Tremelimumab in combination with TACE or RFA in patients with advanced HCC is feasible. Preliminary pathology data will be presented regarding all post-treatment tumor biopsies.

